# Efficient transmission of Cassava brown streak disease viral pathogens by chip bud grafting

**DOI:** 10.1186/1756-0500-6-516

**Published:** 2013-12-06

**Authors:** Henry Wagaba, Getu Beyene, Cynthia Trembley, Titus Alicai, Claude M Fauquet, Nigel J Taylor

**Affiliations:** 1 Donald Danforth Plant Science Center, 975 N. Warson Rd, St Louis, MO, USA; 2 National Crops Resources Research Institute, Namulonge, P.O. Box 7084, Kampala, Uganda; 3 Centro Internacional de Agricultura Tropical, Cali-Palmira, Apartado Aéreo 6713, Cali, Colombia

**Keywords:** Cassava brown streak disease, Cassava brown streak virus, Ugandan cassava brown streak virus, Chip bud graft, Virus transmission, Cassava

## Abstract

**Background:**

Techniques to study plant viral diseases under controlled growth conditions are required to fully understand their biology and investigate host resistance. Cassava brown streak disease (CBSD) presents a major threat to cassava production in East Africa. No infectious clones of the causal viruses, *Cassava brown streak virus* (CBSV) or *Ugandan cassava brown streak virus* (UCBSV) are available, and mechanical transmission to cassava is not effective. An improved method for transmission of the viruses, both singly and as co-infections has been developed using bud grafts.

**Findings:**

Axillary buds from CBSD symptomatic plants infected with virulent isolates of CBSV and UCBSV were excised and grafted onto 6–8 week old greenhouse-grown, disease-free cassava plants of cultivars Ebwanateraka, TME204 and 60444. Plants were assessed visually for development of CBSD symptoms and by RT-PCR for presence of the viruses in leaf and storage root tissues. Across replicated experiments, 70-100% of plants inoculated with CBSV developed CBSD leaf and stem symptoms 2–6 weeks after bud grafting. Infected plants showed typical, severe necrotic lesions in storage roots at harvest 12–14 weeks after graft inoculation. Sequential grafting of buds from plants infected with UCBSV followed 10–14 days later by buds carrying CBSV, onto the same test plant, resulted in 100% of the rootstocks becoming co-infected with both pathogens. This dual transmission rate was greater than that achieved by simultaneous grafting with UCBSV and CBSV (67%), or when grafting first with CBSV followed by UCBSV (17%).

**Conclusions:**

The bud grafting method described presents an improved tool for screening cassava germplasm for resistance to CBSD causal viruses, and for studying pathogenicity of this important disease. Bud grafting provides new opportunities compared to previously reported top and side grafting systems. Test plants can be inoculated as young, uniform plants of a size easily handled in a small greenhouse or large growth chamber and can be inoculated in a controlled manner with CBSV and UCBSV, either singly or together. Disease symptoms develop rapidly, allowing better studies of interactions between these viral pathogens, their movement within shoot and root systems, and how they induce their destructive disease symptoms.

## Findings

### Background

Cassava (*Manihot esculenta* Crantz) is a starchy root crop cultivated throughout the tropics [1], with special importance as a source of carbohydrates and income for millions of smallholder farmers in sub-Saharan Africa [2]. Cassava yields in East Africa are suppressed by numerous diseases, of which Cassava brown streak disease (CBSD) and Cassava mosaic disease (CMD) are the most important [3,4].

CBSD is caused by *Ugandan cassava brown streak virus* (UCBSV) and *Cassava brown streak virus* (CBSV), both of which belong to the family *Potyviridae*, genus *Ipomovirus*[5-7]. CBSD symptoms on infected cassava plants are characterized by yellow chlorosis on the secondary and tertiary veins of older leaves, brown colored lesions on mature stems, and in extreme cases, die-back of younger, green stem tissues. Most importantly, infected plants often develop brown, necrotic lesions within their storage roots that render them inedible and valueless at market [8]. Incidence of CBSD has increased significantly over recent years to reach epidemic levels in East Africa, and is reported to be spreading into Central Africa [4]. As a result, the disease is now considered to be among the most important threats to food security in the tropics [3,9].

A lack of knowledge exists concerning CBSD, including how CBSV and UCBSV move within the host, their interaction within infected plants and how the pathogens cause their economically destructive symptoms. Techniques to improve laboratory and greenhouse-based studies of CBSD are required to increase our understanding of the etiology and biology of the disease and investigate occurrence and mechanisms of resistance in conventional and transgenically modified germplasm [10]. CBSD is transmitted under natural field conditions by whiteflies of the species *Bemisia tabaci* Gennadius *(Hemiptera: Aleyrodidae*). However, use of whitefly vectors to transmit the causal viruses under controlled growth conditions is inefficient, labor intensive and costly. In addition, low transmission rates (22% for CBSV [11]) make this method impractical for use in large-scale experiments. While mechanical transmission of CBSV and UCBSV by sap inoculation to the laboratory model plant *Nicotiana benthamiana* is efficient [12], it is completely ineffective for cassava. Infectious clones are not available for either pathogen, leaving graft inoculation as the only reliable method to transmit CBSV and UCBSV between infected and non-infected cassava plants. Recently, we reported the use of a side veneer grafting system to challenge eight-week-old transgenic cassava plants with UCBSV [10]. Although workable, this technique is cumbersome and time-consuming, requiring growth of the virus source plants and test plants to at least two months of age before grafting can take place, and eight weeks or more for disease symptoms to develop. The 1:1 ratio of scion to rootstock plants needed also restricts the number and size of replicated experiments that can be performed using this method.

We describe here an alternative, efficient and more resource effective inoculation technique based on the use of chip bud grafts to transmit CBSD viruses from infected to non-infected cassava plants, either as single infections or as dual infections with both pathogens.

### Experimental data

**Figure 1:**
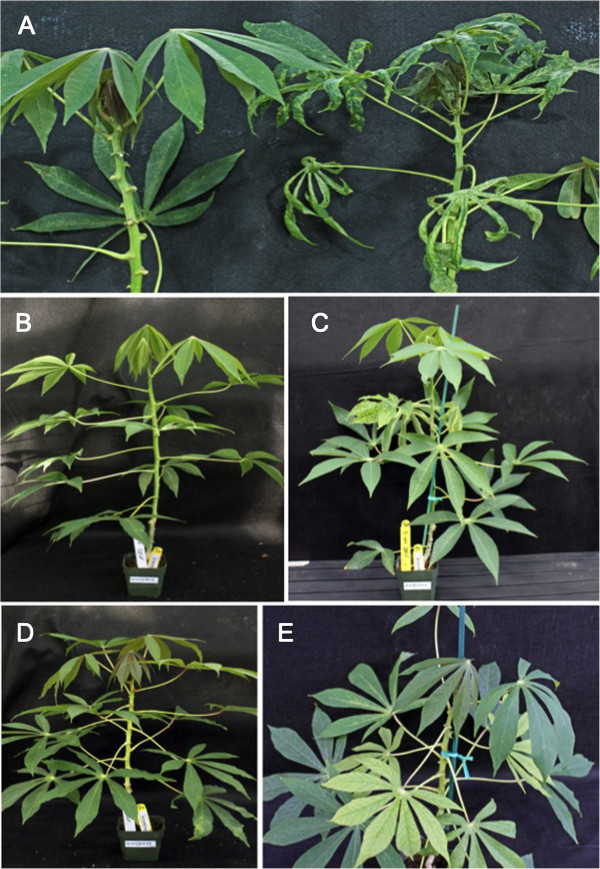
**Disease symptoms on whole cassava plants following transmission by chip bud graft inoculations. (A)** CMD symptoms on cultivar 60444 – non-inoculated plant (left) and plant grafted with a bud carrying EACMV-K201 nine weeks after graft initiation (right) **(B** and **D)** healthy non-inoculated control plants of cultivars TME204 and 60444 at 10 weeks of age and **(C** and **E)** CBSD symptoms on leaves of cultivars TME204 and 60444 at six weeks after grafting with a chip bud carrying CBSV. Leaf symptoms are restricted to leaves within a zone of 4–6 nodes situated 10–12 nodes above the graft site.

**Figure 2:**
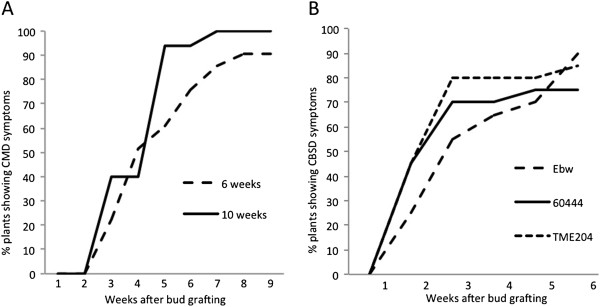
**Development of disease symptoms on shoot tissues of cassava plants after chip bud graft inoculation. (A)** progress in disease incidence in six- and ten-week-old plants of cultivar 60444 after bud graft inoculation with EACMV-K201. Data shown as the average of two independent graft challenge experiments. **(B)** progress in CBSD incidence in cultivars TME204, 60444 and Ebwanateraka (Ebw) after bud graft inoculation with CBSV. Data shown as the average for two independent graft challenge experiments.

As a first step in assessing the ability of chip bud grafts to transmit cassava viruses, axillary buds were excised from plants of cultivar 60444 infected with *East African cassava mosaic virus* (EACMV) isolate K201 and grafted to healthy plants of the same cultivar. EACMV-K201 is a virulent isolate of EACMV causing distinct and severe CMD symptoms on young leaves, and thus acted as indicator of virus transmission by the grafting process. Table 1 shows successful grafting rates when chip buds obtained from plants infected with EACMV (86.7%) and from non-infected plants (95.6%) were grafted to healthy rootstocks. Transmission of CMD at greater than 80% (Table 1) indicated that the chip bud graft method was efficient (Table 1) for transmission of this cassava geminivirus. Figure 1A shows development of CMD with time after bud graft inoculation onto healthy plants of cultivar 60444 for six- and ten-week-old plantlets. No significant differences were seen in the response of plants of different ages to bud graft inoculation. In both cases, CMD symptoms were first seen three to five weeks after grafting and reached a maximum incidence of 75-85% four to eight weeks after grafting (Figures 2A and 1A).

**Table 1:** Transmission of Cassava mosaic disease via bud grafts in cultivar 60444

**Age of test plants**	CMD-free scion buds*	**CMD infected scion buds**^**+**^	
	**Successful grafts/number performed (%)**	**Successful grafts/number performed (%)**	**CMD transmission/total grafts (%)**
Six weeks			
1.	11/12	10/12	9/12
2.	12/12	11/11	10/11
Ten weeks			
1.	9/10	8/10	8/10
2.	11/11	10/12	10/12
Total	43/45 (95.6)	39/45 (86.7)	37/45 (82.2)

*axillary buds were excised from disease free plants and grafted back onto the same mother plant (self graft). Grafting success was assessed one week later and scored positive if the chip buds retained their green color and had attached to the rootstock with callus tissue formed along edges of the graft union.

^**+**^axillary buds were excised from plants infected with EACMV-K201 and grafted onto healthy plants of cv. 60444. CMD transmission was visually assessed and scored for development of typical CMD symptoms on young leaves of the rootstock test plant. Data shown for plants six weeks after grafting.

Data is shown for graft experiments performed twice (1 & 2), at six and ten weeks after plants were established in soil.

**Figure 3:**
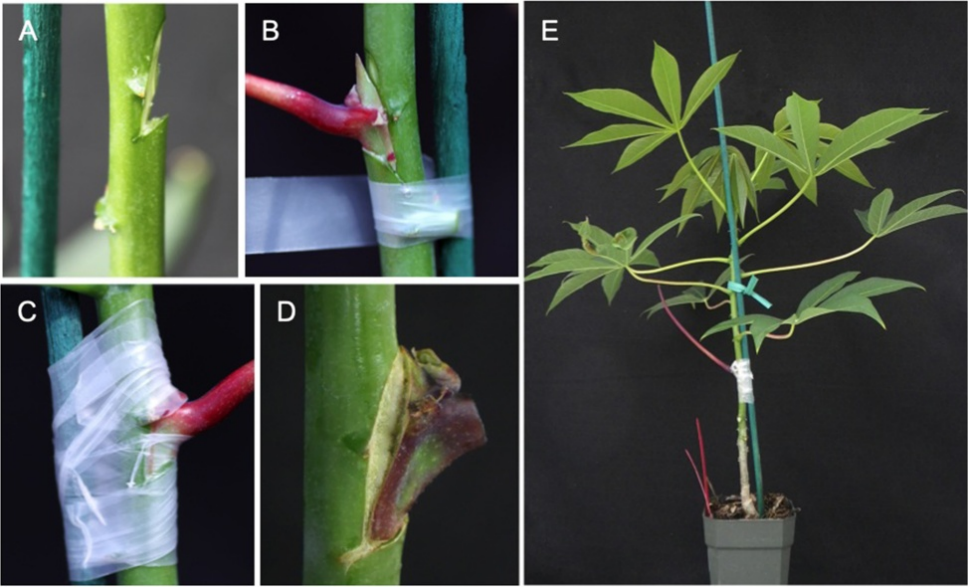
**Steps in chip bud grafting of cassava. (A)** axillary bud is removed from stem portion of six- to eight-week-old rootstock test plant to expose cambium tissue, **(B)** axillary bud excised from non-lignified portion of virus infected plants with petiole attached is inserted into rootstock test plant, **(C)** bud graft secured with parafilm, **(D)** successful bud graft one week after graft initiation with bud remaining healthy and attached to rootstock with visible callus formation and **(E)** whole plant after completion of chip bud grafting.

Having established the ability of bud graft inoculation to transmit CMD, efficiency of CBSD transmission was assessed by grafting buds from cultivar Ebwanateraka plants infected with CBSV onto healthy 6–8 week old rootstocks of cultivars TME204, 60444 and Ebwanateraka. In addition, within each experiment virus-free control grafts were included, whereby CBSD-free buds were excised and grafted back onto the same stem at the same position (self-graft) in order to assess efficiency of the grafting process across the three genetic backgrounds. Table 2 shows success rates for bud grafting and transmission of CBSV as determined by development of CBSD symptoms on stems and leaves. Across the replicated experiments, 100% of virus-free self-grafts had successful graft union, with the scion bud retaining its green color, and visible callus tissue formed along the edges of the graft union within one week (Figure 3D). When CBSV infected buds were grafted onto healthy plants, development of graft unions varied from 70% to 90% with no difference in success rates between the three cultivars studied. From a total of 60 CBSV bud inoculations performed, 47 (78.3%) formed graft unions. However, 52 (86.7%) plants developed CBSD symptoms, indicating that transmission of the pathogen took place within a week after grafting and that successful graft union is not essential for transmission of the disease.

**Table 2:** Transmission of Cassava brown streak virus symptoms via bud grafts

	CBSV-free scion buds*	**CBSV infected scion buds**^**+**^	
**Cultivar**	**Successful grafts/number performed (%)**	**Successful grafts/number performed (%)**	**CBSV transmission/total grafts (%)**
Ebwanateraka			
1.	5/5	7/10	8/10
2.	5/5	9/10	10/10
60444			
1.	5/5	8/10	9/10
2.	5/5	7/10	7/10
TME204			
1.	5/5	9/10	10/10
2.	5/5	7/10	8/10
**Total**	30/30 (100)	47/60 (78.3)	52/60 (86.7)

*axillary buds were excised and grafted back onto the mother plant. Grafting success was assessed one week later and scored positive if the chip buds retained their green color and had attached to the rootstock with callus tissue formed along edges of the graft union.

^**+**^axillary buds were excised from cultivar Ebwanateraka plants infected with the Naliendele isolate of CBSV and grafted onto healthy plants of three different cassava cultivars. Transmission of CBSV was visually assessed for development of CBSD symptoms on leaves and stem of the rootstock test plant. Data is shown for plants six weeks after grafting in experiments performed twice (1 & 2).

**Figure 4:**
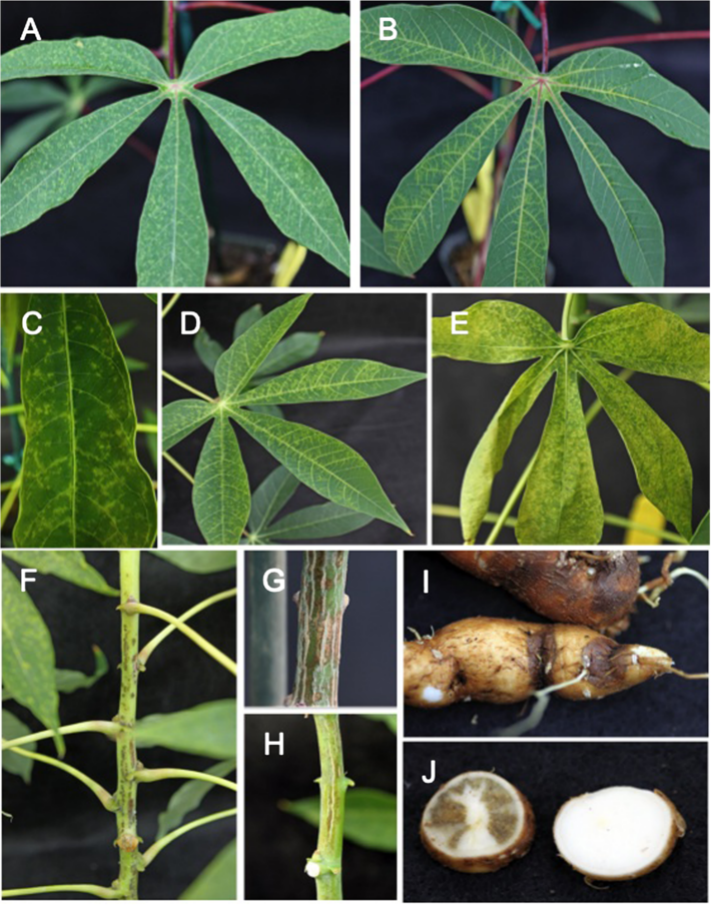
**CBSD symptoms on leaves, stems and storage roots as transmitted by chip bud grafts carrying CBSV. (A)** chlorotic spots seen as first evidence of successful transmission on a leaf of cultivar Ebwanateraka, **(B)** feathery chlorosis on veinal regions of a leaf of cultivar Ebwanateraka, as seen developing on older leaf, **(C)** detail of chlorotic spots on leaf of TME204 3–4 weeks after graft initiation, **(D)** feathery chlorosis on veinal regions of leaf of TME204, **(E)** severe CBSD symptoms on leaf of cultivar TME204 approximately six weeks after graft initiation, **(F)** stem of cultivar 60444 showing severe symptoms of CBSD, **(G)** brown and grey cork lesions forming on stem tissue of CBSV infected cultivar TME204, **(H)** CBSD symptoms visible as brown colored streaks on the stem of cultivar Ebwanateraka, **(I)** brown lesions visible on outer surface of storage roots harvested from plants of cultivar 60444 approximately 2.5 months after chip bud graft inoculation and **(J)** typical CBSD systems seen within the storage parenchyma of tuberous roots 2.5 months after bud graft inoculation (left), symptom-free storage root from non-inoculated plant (right).

Development of CBSD shoot symptoms was scored over time after bud graft inoculation (Figure 2B). Symptoms were first seen on all three cultivars between one and two weeks after grafting, with maximum frequencies of 70-100% CBSD incidence reached within three to four weeks in cultivars TME204 and 60444, and maximum frequencies of 80-90% by five to six weeks after grafting in the case of cultivar Ebwanateraka (Figure 2B). CBSV-induced CBSD leaf symptoms first appeared approximately 10 nodes above the bud graft site as hundreds of small chlorotic spots spread over the entire lamina surface (Figure 4). As these leaves aged, chlorosis became more severe and the leaves often curled upwards along the edges (Figure 4). This pattern was apparent on one or two additional leaves, after which symptoms diminished and then disappeared, with new leaves showing no observable CBSD. Over a subsequent period of 2–3 weeks, feathery chlorosis of the veinal regions developed on one or two leaves below those showing the initial CBSD symptoms described above. As a result, by the end of the six-week observation period, disease symptoms were restricted to leaves within a distinct region of four to six nodes with leaves above and below this remaining asymptomatic (Figure 1C and E).

While development of leaf symptoms was very similar across all three cassava cultivars studied, progression of CBSV-induced stem symptoms differed in timing and appearance. In 60444, stem lesions were first observed at the same time as leaf symptoms. These developed as brown spots and streaks on the stem, at or just above the level of symptomatic leaves, increasing in size with time and becoming pronounced around the nodes and basal regions of the petiole (Figure 4F). In the case of TME204 and Ebwanateraka, stem symptoms became apparent as brown and grey colored streaks and spots from one to two weeks after leaf symptoms and progressed with time up and down the stem as shown in Figure 4G and H.

**Figure 5:**
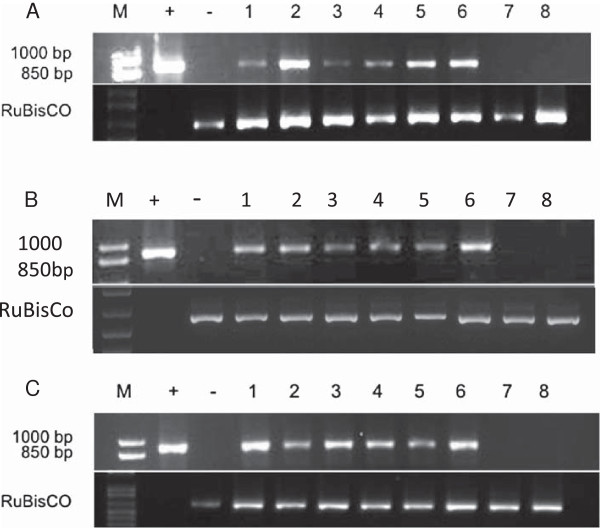
**Reverse transcription polymerase chain reaction (RT-PCR) to detect CBSV in leaves of cassava plants 12 weeks after chip bud inoculation (A) TME204, (B) 60444 and (C) Ebwanateraka.** Specific primers were used to detect 896 bp of the CBSV isolate Naliendele coat protein (CBSV-[TZ:Nal3-1:07]). Lanes are M: 1 kb + molecular marker; +: plasmid positive control; -: self-grafted, non-inoculated negative control; 1–6: CBSD symptomatic chip bud inoculated plants; 7–8: asymptomatic chip bud inoculated plants. Rubisco was run as a control.

RT-PCR was performed on total RNA extracted from the youngest symptomatic leaves of a selection of CBSD symptomatic, asymptomatic and control plants at 12 weeks after grafting. Correlation of visible symptoms and presence of the virus was confirmed in all cases, with all symptomatic plants found to be positive for presence of CBSV, while self-grafted and asymptomatic plants were free of detectable levels of the virus (Figure 5).

At six weeks after graft inoculation, five CBSV infected and control plants were transferred to larger pots and cultivated for a further three months. Over that period CBSD symptoms developed along the stem tissues occasionally as visible feathery, veinal chlorosis on older leaves. At the end of this period the plants were harvested and the tuberous roots examined. Distinct signs of CBSD were visible on storage roots from all plants that were RT-PCR positive for presence of CBSV. These were apparent as dark brown lesions on the outer surface (Figure 4I) and as distinct grey to brown necrotic regions within the storage parenchyma tissues (Figure 4J). No such symptoms were seen on CBSV-free plants.

**Figure 6:**
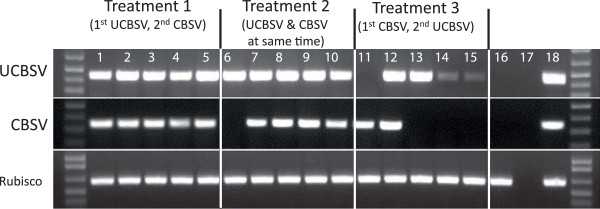
**Reverse transcription polymerase chain reaction (RT-PCR) for the detection of UCBSV (440 bp top panel) and CBSV (344 bp, middle panel) in a dual challenge experiment.** Lane M at each border is 1 kb + molecular marker (Invitrogen), Treatment 1 (samples 1–5), Treatment 2 (sample 6–10) and Treatment 3 (samples 11–15). Lane 16 is a non-grafted plant of 60444, lane 17 is PCR water negative control and lane 18 is a positive control for each virus. Rubisco (bottom panel) was run to demonstrate cDNA quality. Cassava leaves were sampled 7–9 weeks after chip bud inoculation and data shown for one of the two replicated experiments.

The versatility of the bud grafting method was further assessed by attempting co-transmission of both CBSV and UCBSV to the same plant. Three treatments were compared. In the first, plants of cultivar 60444 were grafted simultaneously with two buds, one each carrying CBSV and UCBSV inserted one above the other on the stem of the same test plant. In the second, grafting with a UCBSV-infected bud was followed 10–14 days later with one carrying CBSV and lastly grafting was performed first with CBSV, followed by a bud infected with UCBSV. Efficiency of virus transmission was determined by RT-PCR diagnostics approximately seven weeks after the second grafting. In replicated experiments, all plants (12/12 100%) graft inoculated with UCBSV first followed by CBSV tested positive for both viruses, compared to 8/12 (66.6%) when both viruses were graft inoculated on the same day (Table 3, Figure 6). In contrast, only 2/12 (16.6%) plants tested positive for presence of both viruses when CBSV was graft inoculated, followed by UCBSV. Fourteen weeks after grafting of the second bud, storage roots were harvested. Typical CBSD symptoms (Figure 4I and J) were observed on all co-infected plants and plants RT-PCR positive for presence of CBSV.

**Table 3:** RT-PCR detection of CBSV and UCBSV in leaves of double graft challenged cassava plants of cultivar 60444

	**Number of plants tested positive for both viruses/total grafted**		
**Treatments**	**Experiment 1**	**Experiment 2**	**Total (%)**
**1. UCBSV-CBSV**	4/5	4/7	8/12 (66.6)
**2. UCBSV/CBSV**	5/5	7/7	12/12 (100)
**3. CBSV/UCBSV**	1/5	1/7	2/12 (16.6)

Axillary buds were grafted onto healthy plants of cultivar 60444 either sequentially or at the same time with the two bud inoculum sources as follows: UCBSV and CBSV at the same time (Treatment 1), UCBSV first followed 10–14 days later by CBSV (Treatment 2), and CBSV first followed 10–14 days later by UCBSV (Treatment 3). Data is shown for leaves sampled 7–9 weeks after the second graft inoculation.

### Summary

A chip bud graft method has been developed for efficient transmission of both DNA and RNA viruses from infected to non-infected cassava plants. Graft inoculation systems are the only reliable method of transmitting CBSD viral agents from cassava to cassava under controlled growth conditions. The protocol described here presents numerous improvements over the side-veneer and top wedge grafting methods previously employed for this process [10,13]. In our hands, use of chip bud grafts allows one infected plant to provide nine to ten axillary buds as inoculum for viral challenge experiments, compared to a 1:1 ratio of scion to rootstock in the case of top wedge and side-veneer techniques [14]. Transmission rates of CBSD viruses were efficient (70-100%) via this method and independent of the genetic background of the infected bud scions and test rootstocks. Ability has been shown to efficiently graft challenge rootstock material as early as six weeks old, when plants are relatively small and from like to non-like genetic backgrounds with rapid development of disease symptoms (Figure 1). Bud grafting therefore offers a process for efficient assessment of resistance to this disease across a range of cassava germplasm and on larger-scale experiments than previously possible within the limitations of controlled plant growth facilities.

Under field conditions, it is common for cassava plants to be infected by multiple DNA and RNA viruses [4]. Utilizing the chip grafting system described in this study it was possible to graft inoculate the same plant with isolates of the two different species CBSV and UCBSV. Initial studies reported here indicate that infection first with less virulent UCBSV followed by CBSV 10–14 days later was required to efficiently establish dual infection with both pathogens. The ability to control inoculation of plants with CBSV and UCBSV in this manner, either singly or together, offers new opportunities to study the interaction of these viral pathogens.

An additional benefit of the chip bud inoculation process is the ability to study viral movement and disease progression. In the present study this was apparent when plants were allowed to age past six weeks, with resulting development of disease within the storage root organs (Figure 4). Presently, little is understood concerning the mechanisms controlling movement of CBSD pathogens within the plant and its correlation with symptom development in the shoot and root systems [14,15]. The chip bud method described here therefore presents not only a new, improved tool for screening germplasm for resistance to CBSD viral agents, but also for studying the underlying mechanisms of pathogenicity in this important disease of cassava.

### Experimental procedures

#### Method of production of cassava plant materials and inoculum source

Cassava cultivars Ebwanateraka and TME204 were originally imported as cuttings from Uganda, and planted in soilless compost at the DDPSC (USA) plant growth facility. Imported shoot material was determined to be free of CMD and CBSD viral pathogens by PCR and RT-PCR, respectively as described by Ogwok et al. [16], and subsequently used to establish *in vitro* plantlets [17]. Cultivar 60444 was obtained from the International Institute of Tropical Agriculture (IITA), Ibadan, Nigeria as disease-free *in vitro* plantlets, which were micro-propagated and used to re-establish plants in the soil [10].

Cassava plants of cultivar Ebwanatereka infected with CBSV isolate Naliendele (CBSV[TZ:Nal3-1:07]) obtained from Dr. N.M. Maruthi, NRI, Greenwich (UK), and UCBSV isolate (UCBSV[UG:T04-42:04]) [18] were propagated with stem cuttings and grown in Fafard 51 soilless potting medium (Conrad Fafard, Inc., Agawam, MA). Plants of cultivar 60444 infected with EACMV isolate K201 (EACMV-KE[KE:Msa:K201:02]) [19] by microparticle bombardment were used as the source of CMD inoculum. All plants were grown in Fafard 51 medium in the greenhouse at 28°C and humidity maintained at >50%. Supplemental lighting was provided by 1000 W metal halide light fixtures (HS 2000, P.L. Lighting, Beamsville, ON, Canada) with a day length of 16/8 light/dark cycles.

#### Method for chip bud grafting of cassava plants

Chip bud grafting was performed according to Kester et al. [20]. Axillary buds between 3 mm and 6 mm in width were obtained from non-lignified stems of three-to six-month-old symptomatic plants and used as the source of virus inoculum. Buds with the petiole and leaf attached were excised four to 12 nodes below the apical point from virus-infected and control plants by making a triangular cut with a double-edged razor blade. The bud was excised to a depth of about 2 mm, sufficient to expose the cambium layer (Figure 3A). Axillary buds of equivalent size were excised from the rootstock test plants six to eight nodes above soil level at six to eight weeks after their establishment in soil. The inoculum bud was inserted into and secured to the test plant by wrapping it to the rootstock stem three times with parafilm (Pechiney Plastic Packaging Company, Chicago, IL) (Figure 3B, C and E). The petiole was retained but the leaf blade was removed from the scion bud and grafted plants were returned to the greenhouse where they were maintained under growth conditions as described above. To facilitate challenging of the same test plants with both CBSD causal agents, buds carrying single infections with CBSV or UCBSV were grafted as described above and then with the alternative pathogen two nodes above. Grafting of the two buds done either simultaneously or sequentially within 10–14 days. One week after bud graft insertion the parafilm wrapping was removed, and success or failure of the graft union assessed and recorded. A graft was determined to be successful if the scion bud retained its green color and had fused to the rootstock with visible callus tissue formed at the graft union edges (Figure 3D).

#### Scoring of test plant materials

Plants were visually assessed on a weekly basis on a 1–5 scale [10] for development of CMD or CBSD leaf and stem symptoms beginning one week after grafting. For evaluation of root necrosis due to CBSD, storage roots were also removed from the pots at termination of the experiment 12–14 weeks after planting, and assessed for presence of brown lesions on the outer surface of the peel and sliced open with a razor blade every 2 cm along their length to determine presence of necrotic lesions within the storage parenchyma tissues.

#### RNA extraction and RT-PCR diagnostics

Symptomatic leaves were tested for presence of CBSD viral pathogens by RT-PCR. Leaf samples were removed and placed on dry ice in a 2 ml Fastprep screwcap sampling tube (Phenix Research products, NC USA) containing a grinding bead and stored at -80°C, or used immediately. Total RNA was isolated from 100–150 mg of leaf tissue following the cetyl trimethyl ammonium bromide (CTAB) protocol [21]. One microgram of total RNA was reverse transcribed using the SuperScript® III First-Strand RT-PCR Kit (Life technologies, USA), primed with oligo(dT)25 to obtain cDNA following the manufacturer’s instructions. The PCR reaction consisted of 1 μl cDNA template and 200 nM primers in a total reaction volume of 25 μl, containing 10 mM Tris–HCl (pH 8.6 at 25°C), 50 mM KCl, 1.5 mM MgCl_2_, 25 units/ml Taq DNA Polymerase, 0.2 mM dNTPs, 5% v/v glycerol and 0.05% v/v Tween-20. PCR was carried out at: 94°C for 2 min followed by 35 cycles of 94°C (30 s), 55°C (30 s) and 72°C (60 s) for denaturation, annealing and extension respectively, using primers CBSV_F and CBSV_R; 5′-GAGCAACAAACGGACAAAGGAA-3′ and 5′-TCGATAGCGGCACCCGCGTAG-3′, designed to amplify 896 bp of the CBSV coat protein sequence. For plants challenged with both viruses, a forward degenerate primer CBSVF2 (5′-GGRCCATACATYAARTGGTT-3′) described by Mohammed et al. [14] was used with some modification whereby the degenerate alphabets in the reverse primers were replaced by the actual sequences of the two virus isolates used in this study (5′-CCCTTTGCAAAGCTGAAATAAC-3′ for CBSV; 5′-CCATTATCTCTCCACAGCTTC-3′ for UCBSV). These specific primers can amplify a fragment of about 344 bp in CBSV and a fragment of about 440 bp in UCBSV. PCR amplification was performed separately for each virus to avoid amplification bias. The PCR products were separated on a 1% w/v agarose gel, imaged and recorded using an Alpha Imager™ 2200 (Alpha Innotech Corp, San Leandro, CA, USA).

## Abbreviations

CBSD: Cassava brown streak disease; CBSV: Cassava brown streak virus; CMD: Cassava mosaic disease; EACMV: East African cassava mosaic virus; Ebw: Ebwanateraka; IITA: International institute of tropical agriculture; PCR: Polymerase chain reaction; RT-PCR: Reverse transcription polymerase chain reaction; UCBSV: Ugandan cassava brown streak virus.

## Competing interests

The authors declare that they have no competing interests.

## Authors’ contributions

HW designed the experiments, carried out molecular analysis and co-wrote the manuscript GB planned and performed molecular analysis of the dual challenged plants and co-wrote the manuscript. CT developed the bud grafting method, performed bud grafting, photographed and took care of the plants. TA supervised the experiments and provided the cassava materials CF supervised the experiments and analyzed data. NT designed and supervised the experiments, analyzed data and co-wrote the manuscript. All authors read, contributed to revisions and approved the final manuscript.
